# Parallels in Intercellular Communication in Oomycete and Fungal Pathogens of Plants and Humans

**DOI:** 10.1371/journal.ppat.1003028

**Published:** 2012-12-13

**Authors:** Soo Chan Lee, Jean B. Ristaino, Joseph Heitman

**Affiliations:** 1 Molecular Genetics and Microbiology, Duke University Medical Center, Durham, North Carolina, United States of America; 2 Department of Plant Pathology, North Carolina State University, Raleigh, North Carolina, United States of America; The University of North Carolina at Chapel Hill, United States of America

## Introduction

**Figure 1:**
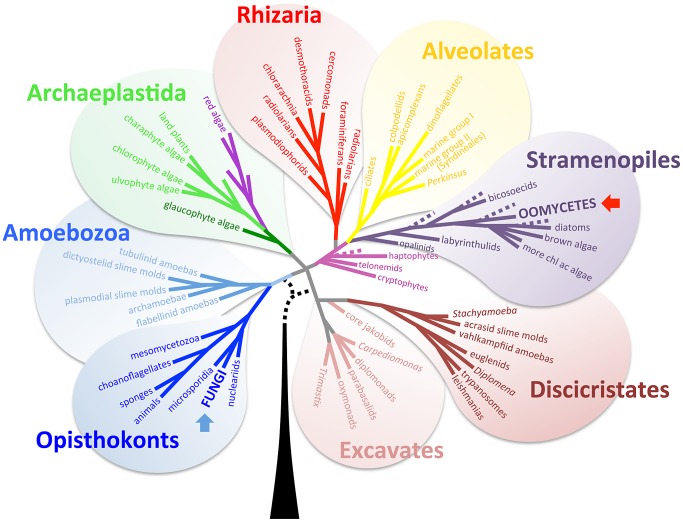
Eukaryotic tree of life (adapted from Baldauf, *Science*, 2003 [**1**], with her permission). The Mucoralean fungi belong to the fungal kingdom (blue arrow) in the Opisthokonts supergroup. *Phytophthora* species belong to the oomycetes in the Stramenopiles supergroup (red arrow).

Sexual reproduction is one of the most fascinating evolutionary outcomes in nature. Sexual development is paradoxical, conferring both benefits and costs, which makes sex an attractive subject in evolutionary biology. In pathogenic microbes, sexual development generates progeny with diverse genetic repertoires and can contribute to create more virulent genotypes. Sexual reproduction is ubiquitous in eukaryotic organisms, from single-celled yeasts to humans. Mating systems are highly adapted in each group and vary from species to species, which results in extremely diverse sexual modes throughout nature. However, in some cases, quite divergent groups share similar mechanisms. This review describes a similarity in pheromone synthesis routes in two group of microbial pathogens of historic importance that are evolutionarily quite distinct: zygomycete pathogenic fungi that belong to the kingdom Fungi in the opisthokonts clade, and *Phytophthora* oomycete species that belong to the stramenopile supergroup [1] (Figure 1).

## Historical Aspects of the Two Evolutionarily Distinct Pathogenic Molds: Mucoralean Fungi and *Phytophthora*

Mucorales of the fungal Zygomycota and *Phytophthora* in the oomycetes have historical significance. One of the *Mucor* species belonging to the Mucoralean order was the first microbe ever observed in detail by the human eye via Robert Hooke's microscope. Hooke described the microscopic structures of this mold in his book *Micrographia* [2]. In addition, the first description of sexual development in fungi was of a Mucoralean species nearly two hundred years ago [3]. Several fungal species in the Mucoralean order are the causal agents of mucormycosis, a deadly fungal infection. These species include *Rhizopus* spp., *Mucor* spp., *Rhizomucor* spp., *Absidia* spp., *Cunninghemella* spp., and others [4]. Mucormycosis is an emerging, serious fungal infection with high mortality rates. A recent mucormycosis outbreak occurred in victims of the tornadoes in Joplin, Missouri, United States.

Oomycetes such as *Phytophthora* spp. were previously considered members of the fungal kingdom. However, more recent molecular analyses revealed oomycetes are not true fungi but instead divergent stramenopiles that are more closely related to the diatoms and brown alga, with only one known human pathogen in the group: *Blastocystis hominis* [5]. *Phytophthora* spp are known as notorious plant destroyers. *Phytophthora infestans* exemplifies this threat; it was the first species described in the genus and left a path of devastation in its wake on potato crops in the US, Ireland, and Europe in the 19th century [6]. Movement of infected potato tubers led to the potato famine epidemics of the 19th century, which resulted in widespread human hunger, disease, and ultimately the death of 2 million people in Ireland. The pathogen is still a threat to food security in the developing world.

## Sexual Development and Mating/*sex* Loci in Mucoralean Fungi

There are two mating types, (+) and (−), in heterothallic Mucoraleans involved in sexual reproduction. When two opposite mating type cells encounter and recognize each other, mating occurs. Upon recognition, hyphae of two different mating types fuse and form a zygophore. This is followed by zygospore formation, in which multiple nuclei exist and diploidization occurs. Meiosis occurs next and the zygospores germinate to produce a sporangium filled with progeny spores. Sexual development in fungi was first described in the Zygomycota, especially in the Mucoralean fungi. However, how sexual reproduction is genetically governed was not known until a series of studies identified the *sex* locus in several Mucoralean fungi, including *Phycomyces blakesleeanus*, *Mucor circinelloides*, *Rhizopus delemar*/*R. oryzae*, *Syzygites megalocarpus*, and *Mucor mucedo* [7]–[11]. Both mating type cells encode allelic HMG transcription factor genes *sexP* and *sexM* for the (+) and (−) mating types, respectively, which function as key transcription factors for mating and cell type identity. The *sexP* and *sexM* genes are flanked by genes encoding a putative RNA helicase and a triose phosphate transporter, thus forming a syntenic TPT-HMG-RNA helicase gene cluster conserved throughout the known *sex* loci in the Mucoraleans.

## Sexual Development and Mating Locus of *Phytophthora*

In heterothallic species of *Phytophthora*, mating occurs when two opposite mating types called A1 and A2 are co-cultured. Each mating type can be dimorphic and capable of producing either male antheridia or female oogonia. A1 mating type cells produce the α1 hormone and A2 mating type cells produce the α2 hormone [12]. Hormones play a key role in partner recognition and sexual development. Sexual development in *Phytophthora* is critical to the generation of novel genotypes that have been exported from their origins in South America and have migrated across the globe, causing epidemics that continue to threaten our food supply today [13]. Understanding mechanisms that trigger sexual reproduction in nature may lead to novel approaches for disease control. Oospores result from sex and can survive for extended periods in soil and plant tissue and cause epidemics earlier in the season.

The complicated genetics of mating have been studied in *P. infestans* and a mating type locus has been identified that exhibits non-Mendelian inheritance [14], [15]. A1 strains are heterozygous and A2 strains are homozygous [16]. Chromosome-specific allele differences and repetitive DNA has been found near the mating type locus in A1 strains and variation in the mating type locus among strains has been reported [17].

## Sexual Pheromone Synthesis in Mucoralean and *Phytophthora*: Evolutionary Convergence

Sexual development is initiated by partner recognition. Molecules, called pheromones, mediate this process. The term “pheromone" (from the Greek words *pherein* [to transport] and *horman* [to stimulate]) was first described and used by Karlson and Lusher one half century ago [18], [19]. Since then, it has become a common term in reference to a sexual development messenger.

**Figure 2:**
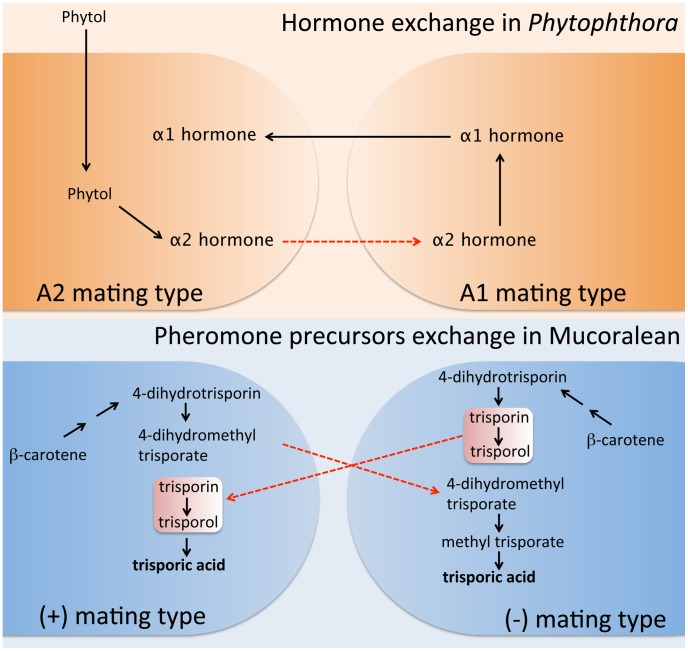
Sexual pheromone synthesis in *Phytophthora* and Mucoralean fungi. In *Phytophthora*, A2 mating type cells produce α2 hormone from phytol. The α2 hormone must be transported into the A1 mating type cells to serve as a precursor of the α1 hormone (upper). In Mucoralean fungi, both mating type cells produce pheromone intermediates from β-carotene. The mating type unique intermediates then must be transported into the opposite mating type partners, where the synthesis of the mature mating pheromone, trisporic acid, is completed (bottom). Thus, in both microbes, pheromone synthesis cannot be completed without mating partners in close proximity. Furthermore, exchange of pheromone intermediates is a key characteristic shared in both evolutionarily distinct pathogens.

Mucoralean fungi utilize trisporic acid as their pheromone. The two mating types collaborate for its production, in which precursors of trisporic acid unique to each mating type need to be transferred into the opposite mating type and are then processed into the final active trisporic acid [20], [21]. In both mating types, β-carotene serves as precursor for trisporic acid production. Both mating type cells convert β-carotene into 4-dihydrotrisporin via several enzymatic reactions. Next, (+) mating type cells produce 4-dihydromethyl trisporate from 4-dihydrotrisporin, whereas (−) mating type cells produce trisporin and trisporol. Interestingly, at this point, the intermediate chemicals are delivered to the opposite mating types. Trisporin and trisporol are exported by (−) mating type cells and enter into (+) mating type cells, where trisporic acid is finally produced. On the other hand, 4-dihydromethyl trisporate is made by (+) mating type cells and then exported and imported into (−) mating type cells, which convert it into methyl trisporate and finally trisporic acid (Figure 2) (reviewed in [22]). Thus, trisporic acid production and sexual development cannot be completed without this chemical dialogue between both of the opposite sex partners.

Remarkably, this collaboration in sexual pheromone production between two fungal mating types is also found in the evolutionarily distant *Phytophthora* oomycete pathogens (Figure 1). The chemical nature of both *Phytophthora* α1 and α2 hormones was recently elucidated [23]–[25]. Only trace amounts of hormone are needed to initiate sexual development, and thus it proved difficult to obtain enough α2 hormone for structural study. In a recent study, Ojuka et al. successfully accumulated enough α2 hormone through a large-scale culture (approximately 200-liter culture) and identified the chemical structure of the α2 hormone [26]. In this study, one of the most interesting findings was that the mechanism of sexual hormone production in *Phytophthora* resembles the intraspecies crosstalk observed in the Mucorales. They demonstrated that the α2 hormone of *Phytophthora* is a precursor of the α1 hormone. α2 hormone production is stimulated by the plant hormone phytol, which is proposed to be a precursor of α2. The α2 hormone from the A2 mating type must be transferred to and converted into α1 hormone by the opposite A1 mating type strain (Figure 2). Subsequently, the α1 hormone diffuses back to the A2 type, inducing gametangia formation. The central role of phytol, a plant-based sterol, in the mating process of *Phytophthora* solves a long standing mystery in mating of this important group of plant pathogens.

Mucoralean and *Phytophthora* are evolutionarily distant in the eukaryotic tree of life (Figure 1), but their sexual pheromone synthesis mechanisms are strikingly parallel. This convergence in exchange of mating pheromone precursors between partners during sexual reproduction illuminates our understanding of the fascinating nuances of sex in nature and potentially provides a foothold to develop new approaches to overcome these divergent pathogens of plants and of humans.
